# Genetic correlation between multiple myeloma and chronic lymphocytic leukaemia provides evidence for shared aetiology

**DOI:** 10.1038/s41408-018-0162-8

**Published:** 2018-12-21

**Authors:** Molly Went, Amit Sud, Helen Speedy, Nicola J. Sunter, Asta Försti, Philip J. Law, David C. Johnson, Fabio Mirabella, Amy Holroyd, Ni Li, Giulia Orlando, Niels Weinhold, Mark van Duin, Bowang Chen, Jonathan S. Mitchell, Larry Mansouri, Gunnar Juliusson, Karin E Smedby, Sandrine Jayne, Aneela Majid, Claire Dearden, David J. Allsup, James R. Bailey, Guy Pratt, Chris Pepper, Chris Fegan, Richard Rosenquist, Rowan Kuiper, Owen W. Stephens, Uta Bertsch, Peter Broderick, Hermann Einsele, Walter M. Gregory, Jens Hillengass, Per Hoffmann, Graham H. Jackson, Karl-Heinz Jöckel, Jolanta Nickel, Markus M. Nöthen, Miguel Inacio da Silva Filho, Hauke Thomsen, Brian A. Walker, Annemiek Broyl, Faith E. Davies, Markus Hansson, Hartmut Goldschmidt, Martin J. S. Dyer, Martin Kaiser, Pieter Sonneveld, Gareth J. Morgan, Kari Hemminki, Björn Nilsson, Daniel Catovsky, James M. Allan, Richard S. Houlston

**Affiliations:** 1 Division of Genetics and Epidemiology, The Institute of Cancer Research, London, SW7 3RP UK; 2 Northern Institute for Cancer Research, Newcastle University, Newcastle upon Tyne, NE2 4HH UK; 3 German Cancer Research Center, 69120 Heidelberg, Germany; 4 Center for Primary Health Care Research, Lund University, SE-205 02 Malmo, Sweden; 5 Division of Molecular Pathology, The Institute of Cancer Research, London, SW7 3RP UK; 6 Myeloma Institute for Research and Therapy, University of Arkansas for Medical Sciences, Little Rock, AR 72205 USA; 7 Department of Internal Medicine V, University of Heidelberg, 69117 Heidelberg, Germany; 8 Department of Hematology, Erasmus MC Cancer Institute, 3075 EA Rotterdam, The Netherlands; 9 Department of Immunology, Genetics and Pathology, Science for Life Laboratory, Uppsala University, 75105 Uppsala, Sweden; 10 Lund Strategic Research Center for Stem Cell Biology and Cell Therapy, Hematology and Transplantation, Lund University, Lund, Sweden; 11 Unit of Clinical Epidemiology, Department of Medicine, Karolinska Institutet, Stockholm, Sweden; 12 Ernest and Helen Scott Haematological Research Institute, Leicester University, Leicester, UK; 13 Department of Haematology, Hull Royal Infirmary, Hull, UK; 14 Hull York Medical School and University of Hull, Hull, UK; 15 Department of Haematology, Birmingham Heartlands Hospital, Birmingham, UK; 16 Department of Haematology, School of Medicine, Cardiff University, Cardiff, UK; 17 Cardiff and Vale National Health Service Trust, Heath Park, Cardiff, UK; 18 National Centre of Tumor Diseases, 69120 Heidelberg, Germany; 19 University Clinic of Würzburg, 97080 Würzburg, Germany; 20 Clinical Trials Research Unit, University of Leeds, Leeds, LS2 9PH UK; 21 Institute of Human Genetics, University of Bonn, D-53127 Bonn, Germany; 22 Division of Medical Genetics, Department of Biomedicine, University of Basel, 4003 Basel, Switzerland; 23 Royal Victoria Infirmary, Newcastle upon Tyne, NE1 4LP UK; 24 Institute for Medical Informatics, Biometry and Epidemiology, University Hospital Essen, University of Duisburg–Essen, Essen, Germany; 25 Institute of Human Genetics, University of Bonn, D-53127 Bonn, Germany; 26 Department of Genomics, Life and Brain Center, University of Bonn, D-53127 Bonn, Germany; 27 Hematology and Transfusion Medicine, Department of Laboratory Medicine, BMC B13, SE-221 84 Lund University, Lund, Sweden; 28 Broad Institute, 7 Cambridge Center, Cambridge, MA 02142 USA

## Abstract

The clustering of different types of B-cell malignancies in families raises the possibility of shared aetiology. To examine this, we performed cross-trait linkage disequilibrium (LD)-score regression of multiple myeloma (MM) and chronic lymphocytic leukaemia (CLL) genome-wide association study (GWAS) data sets, totalling 11,734 cases and 29,468 controls. A significant genetic correlation between these two B-cell malignancies was shown (*R*_g_ = 0.4, *P* = 0.0046). Furthermore, four of the 45 known CLL risk loci were shown to associate with MM risk and five of the 23 known MM risk loci associate with CLL risk. By integrating eQTL, Hi-C and ChIP-seq data, we show that these pleiotropic risk loci are enriched for B-cell regulatory elements and implicate B-cell developmental genes. These data identify shared biological pathways influencing the development of CLL and, MM and further our understanding of the aetiological basis of these B-cell malignancies.

## Introduction

Chronic lymphocytic leukaemia (CLL) and multiple myeloma (MM) are both B-cell malignancies, which arise from the clonal expansion of progenitor cells at different stages of B-cell maturity^1–3^. Evidence for inherited predisposition to CLL and MM comes from the six- and two-fold increased risk of the respective diseases seen in relatives of patients^4^.

Recent genome-wide association studies (GWAS) have transformed our understanding of genetic susceptibility to the B-cell malignancies, identifying 45 CLL^5–8^ and 23 MM risk loci^9–13^. Furthermore, statistical modelling of GWAS data indicates that common genetic variation is likely to account for 34% of CLL and 15% of MM heritability^6,14^. Epidemiological observations on familial cancer risks across the different B-cell malignancies suggest an element of shared inherited susceptibility, especially between CLL and MM^4^.

Linkage disequilibrium (LD) score regression is a method which exploits the feature of a test statistic for a given single nucleotide polymorphism (SNP), whereby that test statistic will incorporate the effects of correlated SNPs^15^. Conventional LD score regression regresses trait *χ*^2^ statistics against the LD score for a given SNP, with the coefficient of the regression line providing an estimate of trait heritability. This method can be modified by instead regressing the product of SNP Z-scores from two traits against the SNP LD score, with the slope providing an estimate of genetic covariance between the two traits^16^. This method can be applied to summary statistics, is not biased by sample overlap and does not require multiple traits to be measured for each individual.

By analysis of GWAS data for MM and CLL and applying cross-trait LD score regression, we have been able to demonstrate a positive genetic correlation between CLL and MM. We find evidence of shared genetic susceptibility at 10 known risk loci and by integrating promoter capture Hi-C (PCHi-C) data, ChIP-seq and expression data we provide insight into the shared biological basis of CLL and MM.

## Methods

### GWAS data sets

The data from six previously reported MM GWAS^9–13^ are summarized in Supplementary Table 1. All these studies were based on individuals of European ancestry and comprised: Oncoarray-GWAS (878 cases 7054 controls) UK-GWAS (2282 cases, 5197 controls), Swedish-GWAS (1714 cases, 10,391 controls), German-GWAS (1508 cases, 2107 controls), Netherlands-GWAS (555 cases, 2669 controls) and US-GWAS (780 cases, 1857 controls).

The data from three previously reported CLL GWAS^8–13^ are summarized in Supplementary Table 2. All these studies were based on individuals of European ancestry and comprised: CLL UK1 (505 cases and 2698 controls), CLL UK2 (1236 cases and 2501 controls) and CLL US (2174 cases and 2682 controls).

### Ethics

Collection of patient samples and associated clinico-pathological information was undertaken with written informed consent and relevant ethical review board approval at respective study centres in accordance with the tenets of the Declaration of Helsinki.

Specifically for the Myeloma-IX trial by the Medical Research Council (MRC) Leukaemia Data Monitoring and Ethics committee (MREC 02/8/95, ISRCTN68454111), the Myeloma-XI trial by the Oxfordshire Research Ethics Committee (MREC 17/09/09, ISRCTN49407852), HOVON65/GMMG-HD4 (ISRCTN 644552890; METC 13/01/2015), HOVON87/NMSG18 (EudraCTnr 2007-004007-34, METC 20/11/2008), HOVON95/EMN02 (EudraCTnr 2009-017903-28, METC 04/11/10), University of Heidelberg Ethical Commission (229/2003, S-337/2009, AFmu-119/2010), University of Arkansas for Medical Sciences Institutional Review Board (IRB 202077), Lund University Ethical Review Board (2013/54), the Norwegian REK 2014/97, and the Danish Ethical Review Board (no: H-16032570).

Specifically, the centres for UK-CLL1 and UK-CLL2 are: UK Multi-Research Ethics Committee (MREC 99/1/082); GEC: Mayo Clinic Institutional Review Board, Duke University Institutional Review Board, University of Utah, University of Texas MD Anderson Cancer Center Institutional Review Board, National Cancer Institute, ATBC: NCI Special Studies Institutional Review Board, BCCA: UBC BC Cancer Agency Research Ethics Board, CPS-II: American Cancer Society, ENGELA: IRB00003888—Comite d’ Evaluation Ethique de l’Inserm IRB #1, EPIC: Imperial College London, EpiLymph: International Agency for Research on Cancer, HPFS: Harvard School of Public Health (HSPH) Institutional Review Board, Iowa-Mayo SPORE: University of Iowa Institutional Review Board, Italian GxE: Comitato Etico Azienda Ospedaliero Universitaria di Cagliari, Mayo Clinic Case-Control: Mayo Clinic Institutional Review Board, MCCS: Cancer Council Victoria’s Human Research Ethics Committee, MSKCC: Memorial Sloan-Kettering Cancer Center Institutional Review Board, NCI-SEER (NCI Special Studies Institutional Review Board), NHS: Partners Human Research Committee, Brigham and Women’s Hospital, NSW: NSW Cancer Council Ethics Committee, NYU-WHS: New York University School of Medicine Institutional Review Board, PLCO: (NCI Special Studies Institutional Review Board), SCALE: Scientific Ethics Committee for the Capital Region of Denmark, SCALE: Regional Ethical Review Board in Stockholm (Section 4) IRB#5, Utah: University of Utah Institutional Review Board, UCSF and UCSF2: University of California San Francisco Committee on Human Research, Women’s Health Initiative (WHI): Fred Hutchinson Cancer Research Center and Yale: Human Investigation Committee, Yale University School of Medicine. Informed consent was obtained from all participants.

The diagnosis of MM (ICD-10 C90.0) in all cases was established in accordance with World Health Organization guidelines. All samples from patients for genotyping were obtained before treatment or at presentation. The diagnosis of CLL (ICD-10-CM C91.10, ICD-O M9823/3 and 9670/3) was established in accordance with the International Workshop on Chronic Lymphocytic Leukaemia guidelines.

### Quality control

Standard quality-control measures were applied to the GWAS^17^. Specifically, individuals with low SNP call rate (<95%) as well as individuals evaluated to be of non-European ancestry (using the HapMap version 2 CEU, JPT/CHB and YRI populations as a reference) were excluded. For apparent first-degree relative pairs, we excluded the control from a case-control pair; otherwise, we excluded the individual with the lower call rate. SNPs with a call rate <95% were excluded as were those with a MAF <0.01 or displaying significant deviation from Hardy–Weinberg equilibrium (*P* < 10^−5^). GWAS data were imputed to >10 million SNPs using IMPUTE2 v4 (for CLL) and IMPUTE2 v2.3 (for MM) software in conjunction with a merged reference panel consisting of data from 1000 Genomes Project^18^ (phase 1 integrated release 3 March 2012) and UK10K^19^. Genotypes were aligned to the positive strand in both imputation and genotyping. We imposed predefined thresholds for imputation quality to retain potential risk variants with MAF >0.01 for validation. Poorly imputed SNPs with an information measure <0.80 were excluded. Tests of association between imputed SNPs and MM were performed under an additive model in SNPTESTv2.5^20^. The adequacy of the case-control matching and possibility of differential genotyping of cases and controls was evaluated using a *Q–Q* plot of test statistics. The inflation *λ* was based on the 90% least-significant SNPs and assessment of *λ*_1000_. Details of SNP QC are provided in Supplementary Table 3 and 4. Four principal components, generated using common SNPs, were included to limit the effects of cryptic population stratification in the US-CLL data set. Eigenvectors for the GWAS data sets were inferred using smartpca (part of EIGENSOFT) by merging cases and controls with phase II HapMap samples.

### Meta-analysis

Meta-analyses were performed using the fixed-effects inverse-variance method using META v1.6^21^. Cochran's *Q-*statistic to test for heterogeneity and the *I*^2^ statistic to quantify the proportion of the total variation due to heterogeneity was calculated.

### LD score regression

To investigate genetic correlation between MM and CLL, we implemented cross-trait LD score regression by Bulik-Sullivan et al.^16^. Using summary statistics from the GWAS meta-analysis we implemented filters as recommended by the authors^16^. Specifically, filtering SNPs to INFO >0.9, MAF >0.01, and harmonizing to Hap Map3 SNPs with 1000 Genomes EUR MAF >0.05, removing indels and structural variants, removing strand-ambiguous SNPs and removing SNPs where alleles did not match those in 1000 Genomes. This was performed by running the munge-sumstats.pr script included with ldsc. We ran ldsc.py, part of the ldsc package, excluding the HLA region. We report heritability estimates on the observed scale. There is no distinction between observed and liability scale genetic correlation for case/control traits^16^.

### Shared risk loci

To identify pleiotropic risk loci, that is genetic loci that influence two traits, we identified SNPs previously reported to be associated with each disease at genome-wide significance (*P* < 5 × 10^−8^), as well as highly correlated variants (*r*^2^ > 0.8) at the 45 and 23 known risk loci for CLL and MM, respectively. Within these correlated variant sets at each locus, we determined how many of the CLL susceptibility loci were associated with MM at region-wide significance after Bonferroni correction for multiple testing (i.e. *P*_adj_ < 0.05/45). We then repeated the process, examining MM susceptibility SNPs in CLL, applying a significance level of *P*_adj_ < 0.05/23. A full list of results is summarized in Supplementary Data File 1 and 2.

### Partitioned heritability

A variation of LD score regression, namely stratified LD score regression, can be used to partition heritability according to different genomic categories. For both MM and CLL we applied stratified LD score regression across the baseline model used in Finucane et al.^22^. We plotted the enrichment of functional categories for each disease- this is defined as proportion heritability divided by the total heritability. We excluded from our plot additional flanking regions around each functional category, which authors designed to allow observation of enrichment of SNP heritability in intermediary regions. A plot of the results is found in Supplementary Figure 1.

### Variant set enrichment

To examine enrichment in specific histone mark binding across shared risk loci, we adapted the method of Cowper-Sal lari et al.^23^. Briefly, for each risk locus, a region of strong LD (defined as *r*^2^ > 0.8 and D′ > 0.8) was determined, and these SNPs were considered the associated variant set (AVS). Publically available ChIP-seq data for 6 histone marks from naive B cells was downloaded from Blueprint Epigenome Project^24^. For each mark, the overlap of the SNPs in the AVS and the binding sites was assessed to generate a mapping tally. A null distribution was produced by randomly selecting SNPs with the same characteristics as the risk-associated SNPs, and the null mapping tally calculated. This process was repeated 10,000 times, and *P*-values calculated as the proportion of permutations where null mapping tally was greater or equal to the AVS mapping tally. An enrichment score was calculated by normalizing the tallies to the median of the null distribution. Thus, the enrichment score is the number of standard deviations of the AVS mapping tally from the median of the null distribution tallies. An enrichment plot for naive B cells is shown in Supplementary Figure 2.

### Cell-type-specific analyses

We considered chromatin mark overlap enrichment for genome-wide significant loci in different cell types using the methodology of Trynka et al.^25^. This approach scores GWAS SNPs based on proximity to chromatin mark and fold-enrichment of respective chromatin mark, assessing significance using a tissue-specific permutation method. We obtained chip-seq data for H3K4me3 from primary blood cells and CLL samples downloaded from Blueprint Epigenome project^24^. In addition, we included in our analysis 4 MM cell lines- KMS11, JJN3, MM1-S and L363 processed as previously described^26^. A heat map of results is shown in Supplementary Figure 3.

### eQTL

eQTL analyses were performed using publicly available whole-blood data downloaded from GTeX^27^. The relationship between SNP genotype and gene expression we carried out using Summary-data-based Mendelian Randomization (SMR) analysis as per Zhu et al.^28^. Briefly, if *b*_*xy*_ is the effect size of *x* (gene expression) on *y* (slope of *y* regressed on the genetic value of *x*), *b*_*zx*_ is the effect of *z* on *x*, and *b*_*zy*_ be the effect of *z* on *y*, *b*_*xy*_ (*b*_*zy*_/*b*_*zx*_) is the effect of *x* on *y*. To distinguish pleiotropy from linkage where the top associated cis-eQTL is in LD with two causal variants, one affecting gene expression the other affecting trait we tested for heterogeneity in dependent instruments (HEIDI), using multiple SNPs in each *cis*-eQTL region. Under the hypothesis of pleiotropy *b*_*xy*_ values for SNPs in LD with the causal variant should be identical. For each probe that passed significance threshold for the SMR test, we tested the heterogeneity in the *b*_*xy*_ values estimated for multiple SNPs in the *cis*-eQTL region using HEIDI.

GWAS summary statistics files were generated from the meta-analysis. For the disease discovery GWAS, we set a threshold for the SMR test of *P*_SMR_ < 2.5 × 10^−5^ corresponding to a Bonferroni correction for the number of probes which demonstrated an association in the SMR test. For all genes passing this threshold we generated plots of the eQTL and GWAS associations at the locus, as well as plots of GWAS and eQTL effect sizes (i.e. input for the HEIDI heterogeneity test). HEIDI test *P*-values <0.05 were considered as reflective of heterogeneity. This threshold is, however, conservative for gene discovery because it retains fewer genes than when correcting for multiple testing. SMR plots for significant eQTLs are shown in Supplementary Figures 4, 5 and a summary of results are shown in Supplementary Table 5.

## Results

### Genetic correlation and heritability

**Fig. 1:**
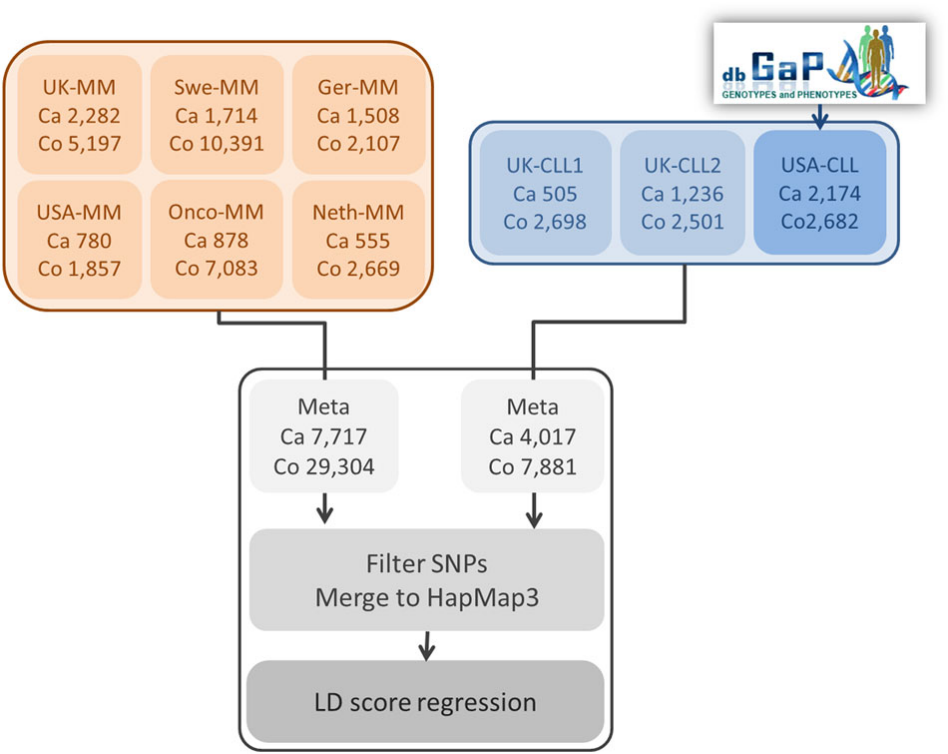
Schematic outlining the processing of data sets used in the genetic correlation

We performed cross-trait LD-score regression using summary statistics from two recent GWAS meta-analyses based on 7717 MM cases and 21,587 controls and 4017 CLL cases and 7881 controls (Fig. 1, Supplementary Table 1-4). While these data sets have been previously subject to quality control (QC)^5–7,9–12^ for the current analysis we implemented additional filtering steps as per Bulik-Sullivan et al.^16^, resulting in 1,055,728 harmonized SNPs between the two data sets. Heritability estimates from cross-trait LD score regression of 9.2 (±1.8%) and 22 (±5.9%) were comparable with previous estimates for MM^14^ and CLL^6^. LD-score regression revealed a significant-positive genetic correlation between MM and CLL with an *R*_g_ value of 0.44 (*P* = 4.6 × 10^−3^).

### Identification of pleiotropic risk loci

**Fig. 2:**
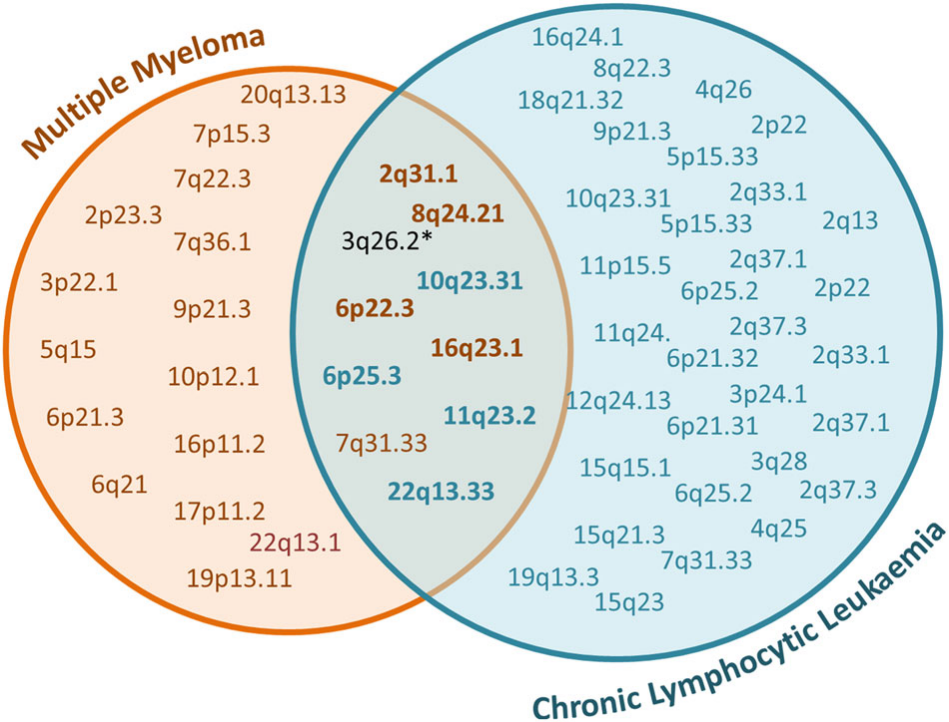
Overlap of loci in multiple myeloma and chronic lymphocytic leukaemia. *correlated variants at 3q26.2 had been previously published as genome wide significant in each data set prior to this analysis

We identified SNPs previously reported to be associated with each disease at genome-wide significance (*P* < 5 × 10^−8^), as well as highly correlated variants (*r*^2^ > 0.8) at the 45 and 23 known risk loci for CLL and MM, respectively. To identify pleiotropic risk loci, that is genetic loci that influence two traits, we determined how many of the CLL susceptibility loci were associated with MM at region-wide significance after Bonferroni correction for multiple testing (i.e. *P*_adj_ < 0.05/45). We then repeated the process, examining MM susceptibility SNPs in CLL, applying a significance level of *P*_adj_ < 0.05/23. Of the 45 CLL risk loci, four were associated with MM (*P*_adj_ < 0.0011) while, of 23 MM risk loci, five were significantly associated in CLL (*P*_adj_ < 0.0022) (Table 1, Fig. 2). Correlated SNPs (*r*^2^ > 0.8) at 3q26.2 are associated with both CLL and MM at genome-wide significance (Fig. 2), bringing the total number of pleiotropic loci to 10.

**Table 1:** Risk loci demonstrating association of alleles at respective loci in both chronic lymphocytic leukaemia (CLL) and multiple myeloma (MM)

Locus	Discovery GWAS	Sentinel variant	Correlated variant	Position (hg19)	Risk allele		Odds Ratio		*P*-value	
					CLL	MM	CLL	MM	CLL	MM
2q31.1	MM	rs4325816		174,808,899	T	T	1.11	1.12	2.0 × 10^−3^	6.4 × 10^−7^
			rs72919402	174,750,200	T	-	1.13	-	4.6 × 10^−4^	-
3q26.2	MM & CLL	rs1317082		169,497,585	A	A	1.20	1.19	7.1 × 10^−8^	2.2 × 10^−16^
			rs3821383	169,489,946	A	A	1.20	1.18	4.2 × 10^−8^	4.5 × 10^−15^
6p25.3	CLL	rs872071		411,064	G	G	1.37	1.10	2.8 × 10^−27^	7.5 × 10^−7^
			rs1050976	408,079	T	T	1.37	1.10	1.9 × 10^−27^	3.7 × 10^−7^
6p22.3	MM	rs34229995		15,244,018	G	G	1.37	1.36	8.5 × 10^−3^	5.6 × 10^−8^
			rs13197919	15,282,334	T	T	1.35	1.32	1.3 × 10^−3^	3.42 × 10^−7^
7q31.33	MM	rs58618031		124,583,896	T	T	1.15	1.11	3.2 × 10^−5^	1.7 × 10^−7^
			rs59294613	124,554,267	C	-	1.16	-	4.4 × 10^−6^	-
8q24.21	MM	rs1948915		128,222,421	C	C	1.17	1.15	7.6 × 10^−7^	2.5 × 10^−12^
			-	-	-	-	-	-	-	-
10q23.31	CLL	rs6586163		90,752,018	A	A	1.28	1.06	1.1 × 10^−16^	1.8 × 10^−3^
			rs7082101	90,741,615	-	C	-	1.06	-	8.2 × 10^−4^
11q23.2	CLL	rs11601504		113,526,853	C	C	1.20	1.09	2.3 × 10^−5^	8.5 × 10^−4^
			-	-	-	-	-	-	-	-
16q23.1	MM	rs7193541		74,664,743	T	T	1.12	1.12	1.0 × 10^−4^	3.7 × 10^−10^
	CLL		-	-	-	-	-	-	-	-
22q13.33		rs140522		50,971,266	T	T	1.17	1.08	3.7 × 10^−7^	1.2 × 10^−4^
			-	-	-	-	-	-	-	-

- indicates SNP not present in filtered data

### Biological inference

Trynka et al. have recently shown that chromatin marks highlighting active regulatory regions overlap with phenotype-associated variants in a cell-type-specific manner^25^. As H3K4me3 was shown to be the most phenotypically cell-type-specific chromatin mark, we examined cell-type specificity of the 10 pleiotropic risk loci by analysing H3K4me3 chromatin marks in normal haematopoietic cells and CLL patient samples from Blueprint, and de novo data on KMS11, MM1S, JJN3 and L363 MM cell lines. Cell types showing the strongest enrichment of risk SNPs at H3K4me3 marks included naive B cells and CD38-B cells. Notably, variants at 2q31.1, 6p25.3, 8q24.21, 16q23.1 and 22q13.33 were enriched for H3K4me3 in naive B cells (Supplementary Figure 3).

Most GWAS signals map to non-coding regions of the genome^29,30^ and influence gene expression through chromatin looping interactions^31,32^. Application of partitioned heritability analysis, stratifying across 53 genomic categories demonstrated enrichment of CLL and MM heritability in functional elements of the genome, in particular FANTOM5 enhancers (CLL and MM) transcription start sites (CLL) and 5′ untranslated region and coding regions (MM) (Supplementary Figure 1). Furthermore, we found significant enrichment of SNPs in the shared loci within regions of active chromatin, as indicated by the presence of H3K27ac and H3K4Me3 marks in naive B cells, supporting the principle that SNPs in shared loci influence risk through regulatory effects (Supplementary Figure 2). To identify target genes we analysed PCHi-C data on naive B cells from Blueprint^24^. We also sought to gain insight into the possible biological mechanisms for associations by performing an expression quantitative trait locus (eQTL) analysis using mRNA expression data on blood from GTEx. Applying Summary data-based Mendelian Randomization (SMR) methodology, we tested for pleiotropy between GWAS signal and *cis*-eQTL for genes to identify a causal relationship. Broadly, our analysis of the shared loci groups them into those which act on a B-cell regulation and differentiation and those which underpin the distinctive biology of cancer; specifically, loci relating to genome instability, angiogenesis and dysregulated apoptosis (Supplementary Table 6).

Of the shared loci, three were related to B-cell regulation. This included composite evidence at 10q23.31, from looping interactions in naive B cells and correlation in GWAS effect size and expression, which provide evidence for two candidate genes *ACTA2*, encoding smooth muscle (α)-2 actin, a protein involved in cell movement and contraction of muscles^33^ and *FAS*, a member of the TNF-receptor superfamily*. FAS*, has a central role in regulating the immune response through apoptosis of B cells^34,35^. At 2q31.1, looping interactions implicated transcription factor *SP3*, which has been shown to influence expression of germinal centre genes,^36,37^. Variants at 6p25.3 reside in the 3′-UTR of *IRF4*, which has an established role in B-cell regulation^38,39^ and MM oncogenesis^40,41^.

Three of the 10 loci contain genes with roles in maintenance of genomic stability. Specifically, evidence from expression and PCHi-C data implicated *RFWD3* at 16q23.1. This gene encodes an E3 ubiquitin-protein ligase, which has been shown to promote progression to late stage homologous recombination through ubiquitination and timely removal of RAD51 and RPA at sites of DNA damage^42^ and is necessary for replication fork restart^43^. Variants in this locus demonstrated enrichment of H3K4me3 marks in two samples of naive B cells, which represents a plausible cell of disease origin. rs58618031 (7q31.33) maps 5′ of *POT1*, the protection of telomeres 1 gene, which is part of the shelterin complex and functions to maintain chromosomal stability^44,45^. Variant rs1317082 at 3q26.2 is located proximal to TERC, a gene which has been shown to influence telomere length^46^. Additionally, we observed looping interactions to a number of genes at 3q26.2 including *SEC62*, which has been proposed as a cancer biomarker^47–50^. Intriguingly, variants at 3q26.2 this locus have been implicated in colorectal^51^, thyroid^52^ and bladder^53^ cancer.

Several genes were implicated at 22q13.33 by looping interactions for *SCO2*, *LMF2, ODF3B, TYMP/ECGF1, NCAPH2*, *SYCE3* and *ARSA*, with *TYMP/ECGF1* and *SCO2* demonstrating evidence of correlation in GWAS and eQTL effect size, albeit not significant after multiple testing (*P*_SMR_ = 2.38 × 10^−4^ and 3.19 × 10^−4^). Variants within this locus were enriched in H3K4me3 chromatin marks in both CD38- B cells and inflammatory macrophages. *TYMP* (alias *ECGF1)* encodes thymidine phosphorylase, which is often overexpressed in tumours and has been linked to angiogenesis^54,55^. A detailed study on this gene has implicated *TYMP* in the development of lytic bone lesions in MM, via a mechanism involving activation of PI3K/Akt signalling and increased *DNMT3A* expression resulting in hypermethylation of *RUNX2*, osterix, and *IRF8*^56^. Furthermore, *SCO2* (synthesis of cytochrome c oxidase), also mapping to this locus, has been implicated in the development of breast^57,58^, gastric^59^ and leukaemia^60^, through glucose metabolism reprogramming^61^, a hallmark of cancer^62^. Tumour suppressor, p53, regulates metabolic pathways, p53-transactivated TP53-induced glycolysis (TIGAR), and regulation of apoptosis in part through SCO2^58,59,61^.

Finally, whereas these data were indifferent to decipher 8q24.21, this locus has also been shown to harbour risk SNPs for other cancers, which localize within distinct LD blocks and likely reflect tissue-specific effects on cancer risk through regulation of MYC^30^.

## Discussion

Our analysis provides evidence of a genetic correlation between MM and CLL. Furthermore, we have identified shared genetic susceptibility at 10 known risk loci. While requiring biological validation, integration of data from PCHi-C, chromatin mark enrichment and eQTL at shared loci has provided insight into how these loci may confer susceptibility to both CLL and MM. Applying a working hypothesis that the loci may act in pleiotropic fashion, we selected relevant cells representing a common tissue of disease origin; namely naive B cells.

A significant genetic correlation between MM and CLL, as well as the discovery of risk loci shared between them, supports epidemiological data demonstrating elevated familial risks between these B-cell malignancies^4^. Furthermore, the shared loci we identified could be broadly grouped into those containing genes related to B-cell regulation and differentiation and those containing genes involved in angiogenesis, genome stability and apoptosis, supporting the tenet that these alleles can influence aetiology of either disease. With the expansion of GWAS of the B-cell malignancies, more detailed characterisation of common underlying risk alleles and affected pathways can inform the biology of B-cell oncogenesis.

## Supplementary information

Supplementary Information

Supplementary Data

## Data Availability

SNP genotyping data that support the findings of this study have been deposited in Gene Expression Omnibus with accession codes GSE21349, GSE19784, GSE24080, GSE2658 and GSE15695; in the European Genome-phenome Archive (EGA) with accession code EGAS00000000001; in the European Bioinformatics Institute (Part of the European Molecular Biology Laboratory) (EMBL-EBI) with accession code E-MTAB-362 and E-TABM-1138; and in the database of Genotypes and Phenotypes (dbGaP) with accession code phs000207.v1.p1. The remaining data are contained within the paper and Supplementary Files or available from the author upon request. Naive B-cell HiC data used in this work is publicly available from Blueprint Epigenome Project [https://osf.io/u8tzp/]. ChIP-seq data for H3K27ac, H3K4Me1, H3K27Me3, H3K9Me3, H3K36Me3 and H3K27Me3 from naive B cells are publicly available and was obtained from Blueprint Epigenome Project [http://www.blueprint-epigenome.eu/].
