# Supplementary material for: Genetic correlation between multiple myeloma and chronic lymphocytic leukaemia provides evidence for shared aetiology
Source: Blood Cancer J. 2018 Dec 21;9(1):1. doi: 10.1038/s41408-018-0162-8 (PMC6315026; doi:10.1038/s41408-018-0162-8)
Supplement: Supplementary file 1 — Supplementary Information [file 41408_2018_162_MOESM1_ESM.docx]

**SUPPLEMENTARY INFORMATION**

**Genetic correlation between multiple myeloma and chronic lymphocytic leukaemia provides evidence for shared aetiology**

Went *et al*

|  | **UK** | | **Sweden/Norway** | | **Germany** | | **Netherlands** | | **USA** | | **OncoArray** | |
| --- | --- | --- | --- | --- | --- | --- | --- | --- | --- | --- | --- | --- |
|  | **Cases** | **Controls** | **Cases** | **Controls** | **Cases** | **Controls** | **Cases** | **Controls** | **Cases** | **Controls** | **Cases** | **Controls** |
|  |  |  |  |  |  |  |  |  |  |  |  |  |
|  |  |  |  |  |  |  |  |  |  |  |  |  |
| Pre-QC | 2,329 | 5,199 |  |  | 1,512 | 2,107 | 608 | 2,669 | 1,076 | 2,234 | 931 | 7,519 |
|  |  |  |  |  |  |  |  |  |  |  |  |  |
| Sex discrepancy | 10 | 0 |  |  | 1 | 0 | 0 | 0 | 0 | 0 | 6 | 8 |
| Call rate fail | 1 | 0 |  |  | 0 | 0 | 2 | 0 | 0 | 4 | 1 | 1 |
| Heterozygosity rate | NA | NA |  |  | NA | NA | 7 | 0 | 9 | 2 | 5 | 7 |
| Related Individuals | 2 | 2 |  |  | 0 | 0 | 0 | 0 | 1 | 0 | 3 | 68 |
| Non-European Ancestry | 34 | 0 |  |  | 3 | 0 | 44 | 0 | 286 | 369 | 44 | 364 |
|  |  |  |  |  |  |  |  |  |  |  |  |  |
| Post-QC | 2,282 | 5,197 | 1,714 | 10,391 | 1,508 | 2,107 | 555 | 2,669 | 780 | 1,857 | 878 | 7,083 |

**Supplementary Table 1:** **Details of the quality control filters applied to each MM GWAS.** Samples were excluded due to call rate (<95% or failed genotyping), ancestry (principle components analysis or other samples reported to be not of white, European descent), relatedness (any individuals found to be duplicated or related within or between data sets through IBS) or sex discrepancy. These studies have been previously reported in their entirety with comprehensive details on QC.

|  | **UK 1** | | **UK 2** | | **US** | |
| --- | --- | --- | --- | --- | --- | --- |
|  | **Cases** | **Controls** | **Cases** | **Controls** | **Cases** | **Controls** |
|  |  |  |  |  |  |  |
|  |  |  |  |  |  |  |
| Pre-QC | 517 | 2,698 | 1,403 | 2,501 | 2,178 | 2,685 |
|  |  |  |  |  |  |  |
| Sex discrepancy |  |  |  |  | 1 | 3 |
| Call rate fail |  |  |  |  | 0 | 0 |
| Heterozygosity rate |  |  |  |  | 0 | 0 |
| Related Individuals |  |  |  |  | 3 | 0 |
| Non-European Ancestry |  |  |  |  | 0 | 0 |
|  |  |  |  |  |  |  |
| Post-QC | 505 | 2,698 | 1,236 | 2,501 | 2,174 | 2,682 |

**Supplementary Table 2:** **Details of the quality control filters applied to each CLL GWAS.** Samples were excluded due to call rate (<95% or failed genotyping), ancestry (principle components analysis or other samples reported to be not of white, European descent), relatedness (any individuals found to be duplicated or related within or between data sets through IBS) or sex discrepancy. These studies have been previously reported in their entirety with comprehensive details on QC.

| **MM** | **UK** | **Sweden/Norway** | **Germany** | **Netherlands** | **USA** | **OncoArray** |
| --- | --- | --- | --- | --- | --- | --- |
|  |  |  |  |  |  |  |
|  |  |  |  |  |  |  |
| Pre-QC | 409,429 |  | 401,405 | 646,124 | 296,998 | 459,068 |
| Call rate fail | 997 |  | 113 | 6,523 | 4 | 6,851 |
| HWE fail/ | 10 |  | 1 | 18,104 | 9,322 | 73,251 |
| Post-QC | 408,422 |  | 401,291 | 621,497 | 287,672 | 378,966 |
| Imputed (filtered) | 8,517,071 | 7,182,761 | 8,282,831 | 8,628,799 | 8,085,846 | 3,874,958 |

**Supplementary Table 3:** **Details of the quality control filters applied to each MM GWAS**. For the OncoArray genotyped SNPs with a call rate <95% were excluded as were those with a MAF <0.01 or showing significant deviation from Hardy-Weinberg equilibrium (i.e. P < 10-5). Imputed SNPs with information score <0.8 and MAF <0.01 were excluded.

| **CLL** | **UK1** | **UK2** | **US** |
| --- | --- | --- | --- |
|  |  |  |  |
|  |  |  |  |
| Pre-QC |  |  | 727,545 |
| Call rate fail |  |  | 2,388 |
| HWE fail/MAF < 0.01 |  |  | 81,128 |
| Post-QC | 301,786 | 630,366 | 644,029 |
| Imputed (filtered) |  |  | 8,899,686 |

**Supplementary Table 4**: **Details of the quality control filters applied to each CLL GWAS**. For the OncoArray genotyped SNPs with a call rate <95% were excluded as were those with a MAF <0.01 or showing significant deviation from Hardy-Weinberg equilibrium (i.e. P < 10-5). Imputed SNPs with information score <0.8 and MAF <0.01 were excluded

|  | |  | |  | |  | **CLL** | | **MM** | | |
| --- | --- | --- | --- | --- | --- | --- | --- | --- | --- | --- | --- |
| **Locus** | **Pos** | | **SNP** | | **Gene** | ***P*_SMR_** | | ***P*_HEIDI_** | | ***P*_SMR_** | ***P*_HEIDI_** |
| 10q23.31 | 90,752,018 | | rs6586163 | | ACTA2 | 1×10^-11^ | | 0.2 | | 3×10^-3^ | 0.5 |
|  |  | |  | | FAS | 6×10^-6^ | | 2×10^-5^ | | 2×10^-3^ | 0.02 |
|  |  | |  | |  |  | |  | |  |  |
| 16q23.1 | 74,664,743 | | rs7193541 | | RFWD3 | 9×10^-3^ | | 0.4 | | 1×10^-6^ | 0.005 |
|  |  | |  | |  |  | |  | |  |  |
| 22q13.33 | 50,971,266 | | rs140522 | | SCO2 | 1×10^-4^ | | 5×10^-6^ | | 3×10^-4^ | 2×10^-4^ |
|  |  | |  | | TYMP | 7×10^-5^ | | 0.03 | | 2×10^-4^ | 0.2 |
|  |  | |  | | ODF3B | 5×10^-5^ | | 0.1 | | 0.01 | 0.3 |

**Supplementary Table 5: Summary of results from SMR analysis.** We set a threshold for the SMR test of *P_SMR_* <2.5×10^-5^ corresponding to a Bonferroni correction for 2000 probes. For all genes passing this threshold we generated plots of the eQTL and GWAS associations at the locus, as well as plots of GWAS and eQTL effect sizes (i.e. corresponding to input for the HEIDI heterogeneity test). HEIDI test *P* values <0.05 were considered as being reflective of heterogeneity. This threshold is conservative for gene discovery because it retains fewer genes than when correcting for multiple testing. Probes which passed the HEIDI threshold are highlighted in grey.

| **Locus** | **Chr** | **rsID** | **Position  (hg19)** | **Functional Evidence** | | | | |
| --- | --- | --- | --- | --- | --- | --- | --- | --- |
|  |  |  |  | **Proximal genes** | **Naïve B HiC** | **eQTL** | **Naïve B Histone Marks present** | **Candidate causal gene(s)** |
| 2q31.1 | 2 | rs4325816 | 174,808,899 | ***SP3*** | ***SP3 (promoter)*** *RP11-394I13.2* |  | H3K27ac+ H3K4me3 | *SP3* |
|  |  |  |  |  |  |  |  |  |
| 3q26.2 | 3 | rs1317082 | 169,497,585 | *ACTRT3  MYNN LRRC34 TERC* | *PDCD10  SERPINI1  RP11-379K17.4  SEC62  SEC62-AS1  GPR160  RNU4-38P  PHC3  RNU6-315P  NA  SKIL  MYNN  SAMD7* |  | H3K27ac+ H3K4me3 H3K27ac+ H3K4me1 | *SEC62*  *TERC* |
|  |  |  |  |  |  |  |  |  |
| 6p25.3 | 6 | rs872071 | 411,064 | *SERPINB6* | *DUSP22 RP3-416J7.5* |  | H3K27ac (weak)+ H3K4me1 |  |
|  |  |  |  |  |  |  |  |  |
| 6p22.3 | 6 | rs34229995 | 15,244,018 | *JARID2* |  |  | H3K27ac (weak)+ H3K4me1 |  |
|  |  |  |  |  |  |  |  |  |
| 7q31.33 | 7 | rs58618031 | 124,583,896 | *POT1 IQUB  ASB15  WASL  ACTRT3  RNU6-11P* | *IQUB  ASB15  RP11-390E23.6  WASL  ACTRT3  RP11-816J6.3 RNU6-11P* |  |  |  |

| **Locus** | **Chromosome** | **rsID** | **Position  (hg19)** | **Functional Evidence** | | | | |
| --- | --- | --- | --- | --- | --- | --- | --- | --- |
|  |  |  |  | **Proximal genes** | **Naïve B HiC** | **eQTL** | **Naïve B Histone Marks present** | **Candidate causal gene(s)** |
| 8q24.21 | 8 | rs1948915 | 128,222,421 |  |  |  | H3K27ac + H3K4me1 |  |
|  |  |  |  |  |  |  |  |  |
| 10q23.31 | 10 | rs6586163 | 90,752,018 | *ACTA FAS* | *ACTA2 (promoter)  FAS (promoter)  CH25H* | *ACTA FAS* | H3K27ac (weak)+ H3K4me3 | *FAS ACTA2* |
|  |  |  |  |  |  |  |  |  |
| 11q23.2 | 11 | rs11601504 | 113,526,853 |  |  |  |  |  |
|  |  |  |  |  |  |  |  |  |
| 16q23.1 | 16 | rs7193541 | 74,664,743 | ***RFWD3*** *GLG1* | *GLG1  RNU6-237P  NPIPB15  AC009120.4  PSMD7  GABARAPL2  TERF2IP  KARS  CFDP1* ***RFWD3 (promoter)*** *RP11-144N1.1  HSPE1P7* | ***RFWD3*** | H3K27ac+ H3K4me3 | *RFWD3* |
|  |  |  |  |  |  |  |  |  |
| 22q13.33 | 22 | rs140522 | 50,971,266 | *TYMP* | *CTA-384D8.36  NCAPH2  ODF3B  SCO2  TYMP  LMF2  NCAPH2  SYCE3  ARSA* | *TYMP ODF3B* |  | *TYMP* |

**Supplementary Table 6: Functional evidence at each of the shared loci**

**Supplementary Figure 1: Partitioned heritability analysis showing results for 28 functional categories.** We used the full baseline model in our analysis as per Finucane *et al^1^,* excluding category flanking regions from our plot.

**
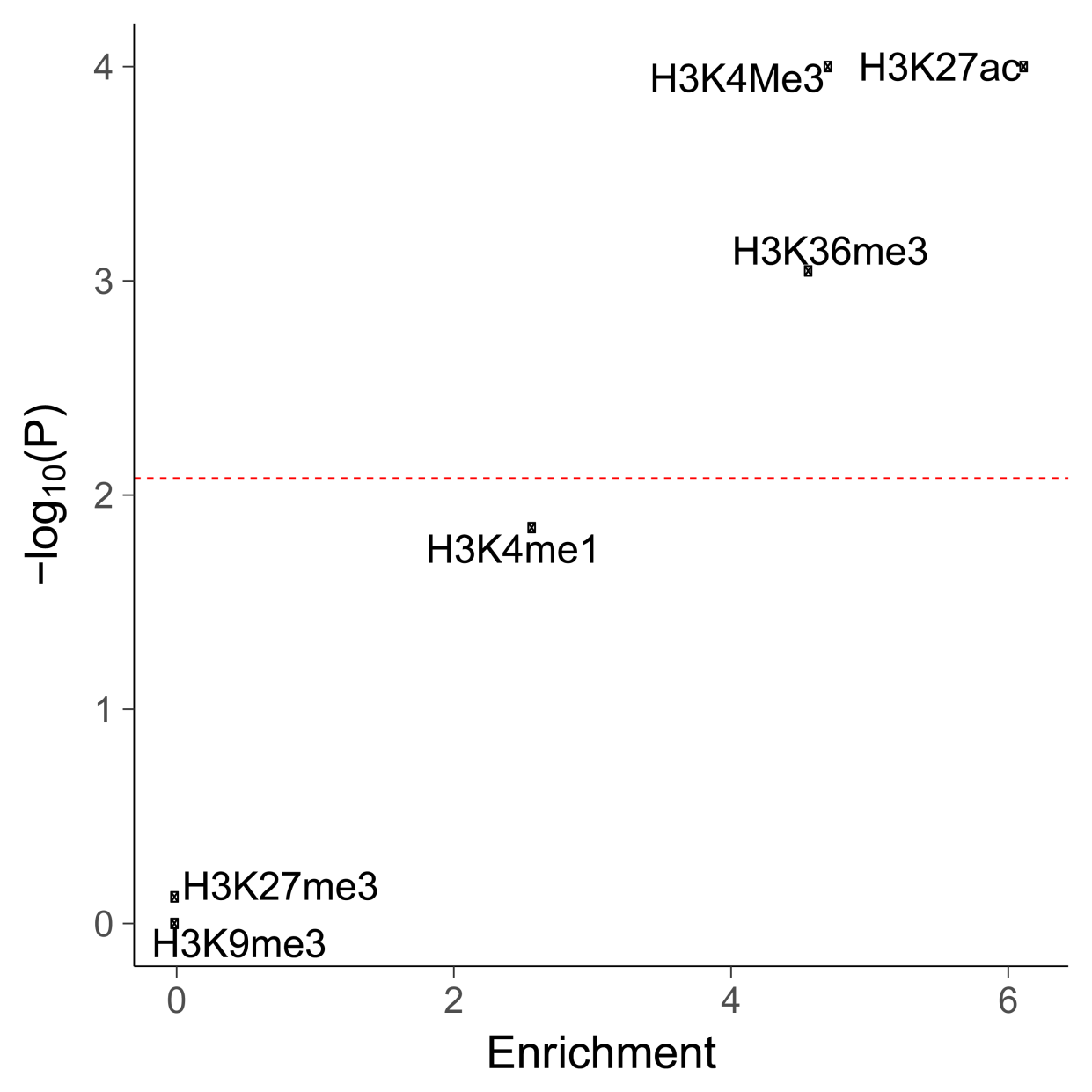
Supplementary Figure 2: The overrepresentation of histone marks in naïve B shared CLL and MM risk SNPs demonstrates that shared risk SNPs are enriched in regions of open chromatin.** The red line denotes the Bonferroni corrected *P*-value threshold.

|  | rs11601504 | rs1317082 | rs140522 | rs1948915 | rs34229995 | rs4325816 | rs58618031 | rs6586163 | rs7193541 | rs872071 |
| --- | --- | --- | --- | --- | --- | --- | --- | --- | --- | --- |
| VB naïve B cell | 0.00 | 0.03 | 0.09 | 0.11 | 0.03 | 0.05 | 0.00 | 0.03 | **0.40** | 0.09 |
| Tonsil naïve B cell | 0.00 | 0.02 | **0.10** | **0.15** | 0.00 | 0.03 | 0.00 | 0.01 | 0.02 | **0.08** |
| VB CD38- naïve B cell | 0.00 | 0.01 | **0.04** | 0.00 | 0.00 | **0.11** | 0.00 | 0.01 | **0.04** | 0.00 |
| KMS11 | 0.00 | 0.00 | 0.04 | 0.00 | **0.45** | 0.01 | **0.99** | 0.01 | 0.01 | 0.00 |
| Thymus CD3+ CD4+ CD8++ thymocyte | 0.00 | **0.30** | 0.02 | 0.00 | 0.00 | 0.03 | 0.00 | 0.02 | 0.02 | 0.00 |
| VB inflammatory macrophage | 0.00 | 0.00 | **0.05** | 0.00 | 0.01 | 0.03 | 0.00 | **0.19** | 0.00 | 0.00 |
| VB mature neutrophil | 0.00 | 0.03 | 0.12 | 0.00 | **0.75** | 0.16 | 0.00 | 0.03 | 0.00 | 0.00 |
|  | 11q23.2 | 3q26.2 | 22q13.33 | 8q24.21 | 6p22.3 | 2q31.1 | 7q31.33 | 10q23.31 | 16q23.1 | 6p25.3 |
|  | TMPRSS5 | MYNN | NCAPH2 |  | JARID2 | SP3 | POT1 | FAS | RFWD3 GLG1 | IRF4 |

**Supplementary Figure 3: Tissue specific H3K4me3 mark enrichment for shared loci.**  Bold denotes SNPs significantly enriched.


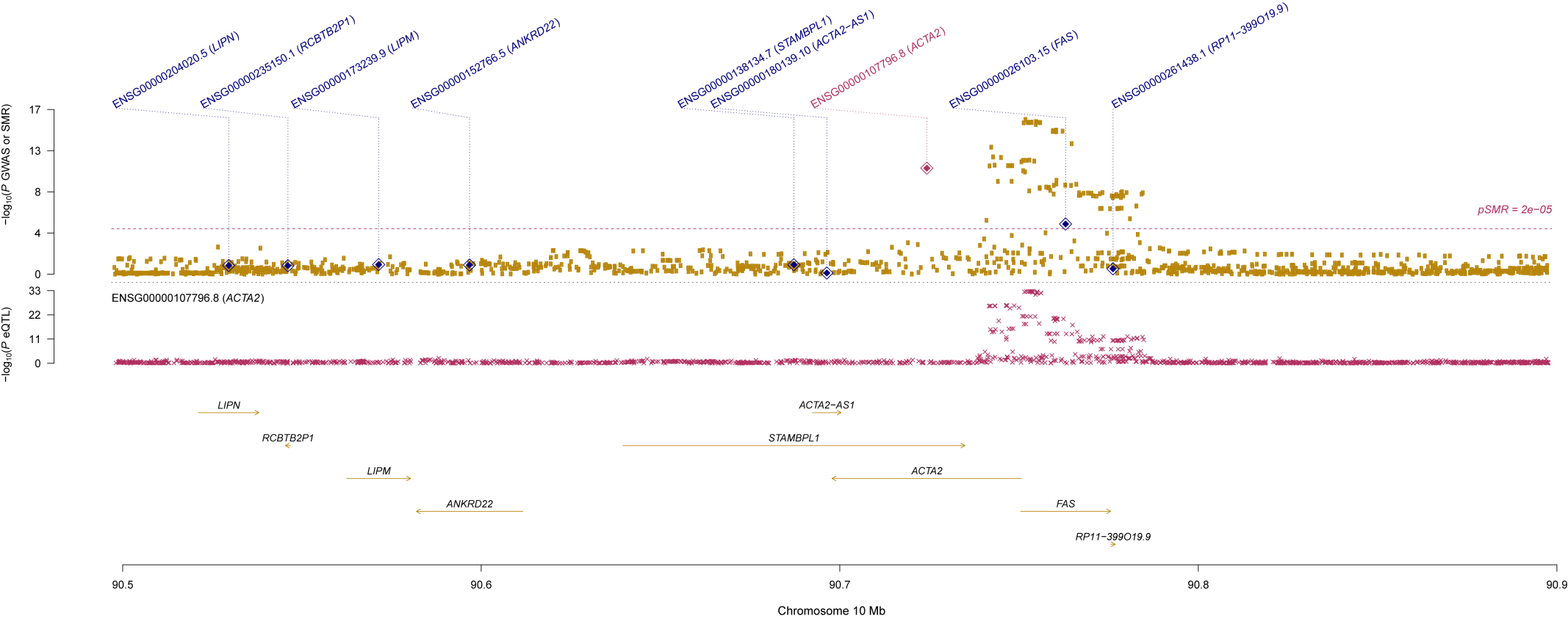
**Supplementary Figure 4a: Summary data-based Mendelian Randomization (SMR) analysis locus plot for chronic lymphocytic leukaemia.** Upper panel - brown dots represent *P*-values for SNPs from the GWAS meta-analysis, diamonds represent *P-*values for probes from the SMR test; lower panel – crosses represent eQTL *P*-values of SNPs from whole blood with genes passing the SMR (i.e. *P_SMR_* < 0.001) and HEIDI (*i.e*. *P_HEIDI_* > 0.05) tests highlighted in red.


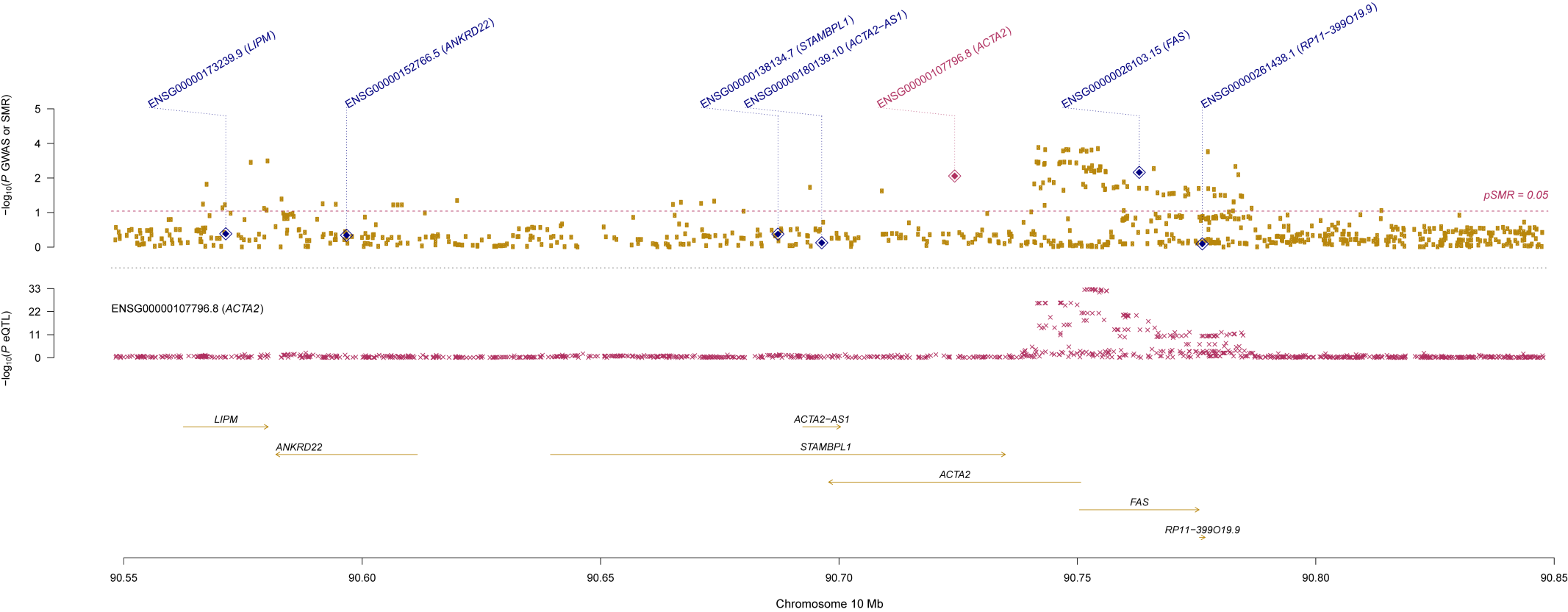


**Supplementary Figure 4b: Summary data-based Mendelian Randomization (SMR) analysis locus plot for multiple myeloma.** Upper panel - brown dots represent *P*-values for SNPs from the GWAS meta-analysis, diamonds represent *P-*values for probes from the SMR test; lower panel – crosses represent eQTL *P*-values of SNPs from whole blood with genes passing the SMR (i.e. *P_SMR_* < 0.001) and HEIDI (*i.e*. *P_HEIDI_* > 0.05) tests highlighted in red.


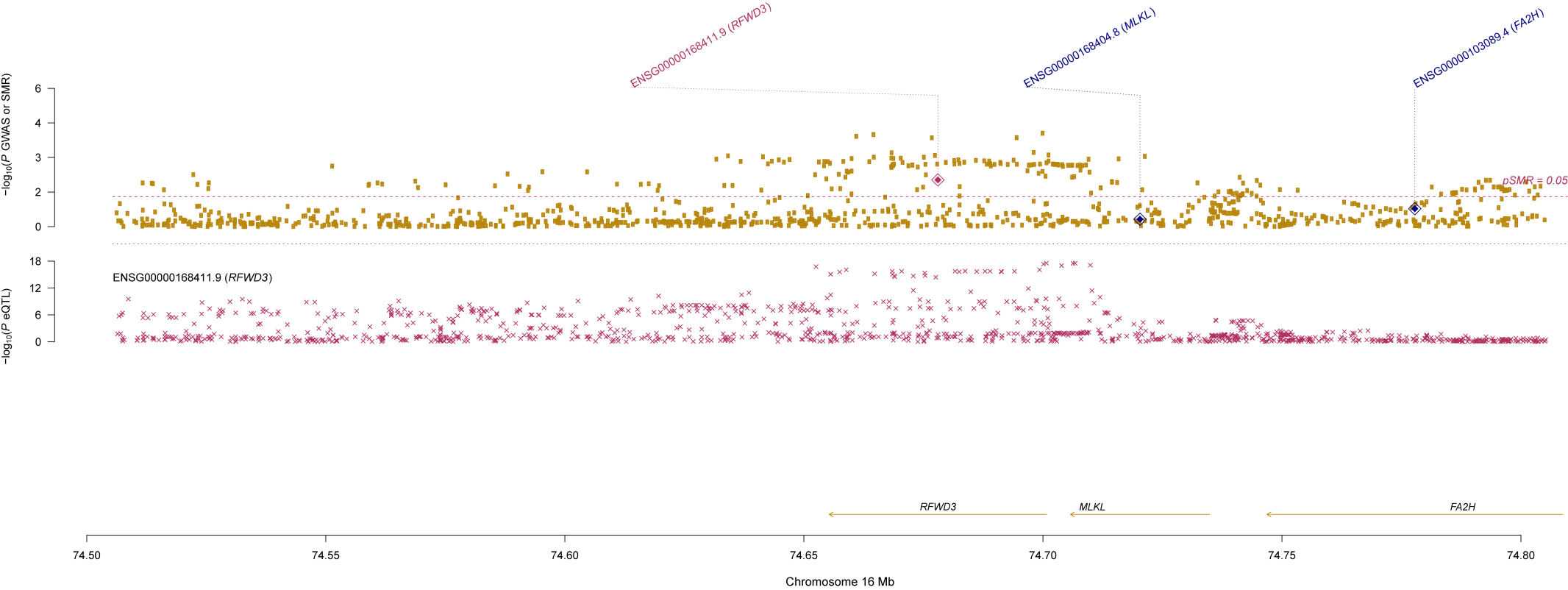


**Supplementary Figure 4c: Summary data-based Mendelian Randomization (SMR) analysis locus plot for chronic lymphocytic leukaemia.** Upper panel - brown dots represent *P*-values for SNPs from the GWAS meta-analysis, diamonds represent *P-*values for probes from the SMR test; lower panel – crosses represent eQTL *P*-values of SNPs from whole blood with genes passing the SMR (i.e. *P_SMR_* < 0.001) and HEIDI (*i.e*. *P_HEIDI_* > 0.05) tests highlighted in red.


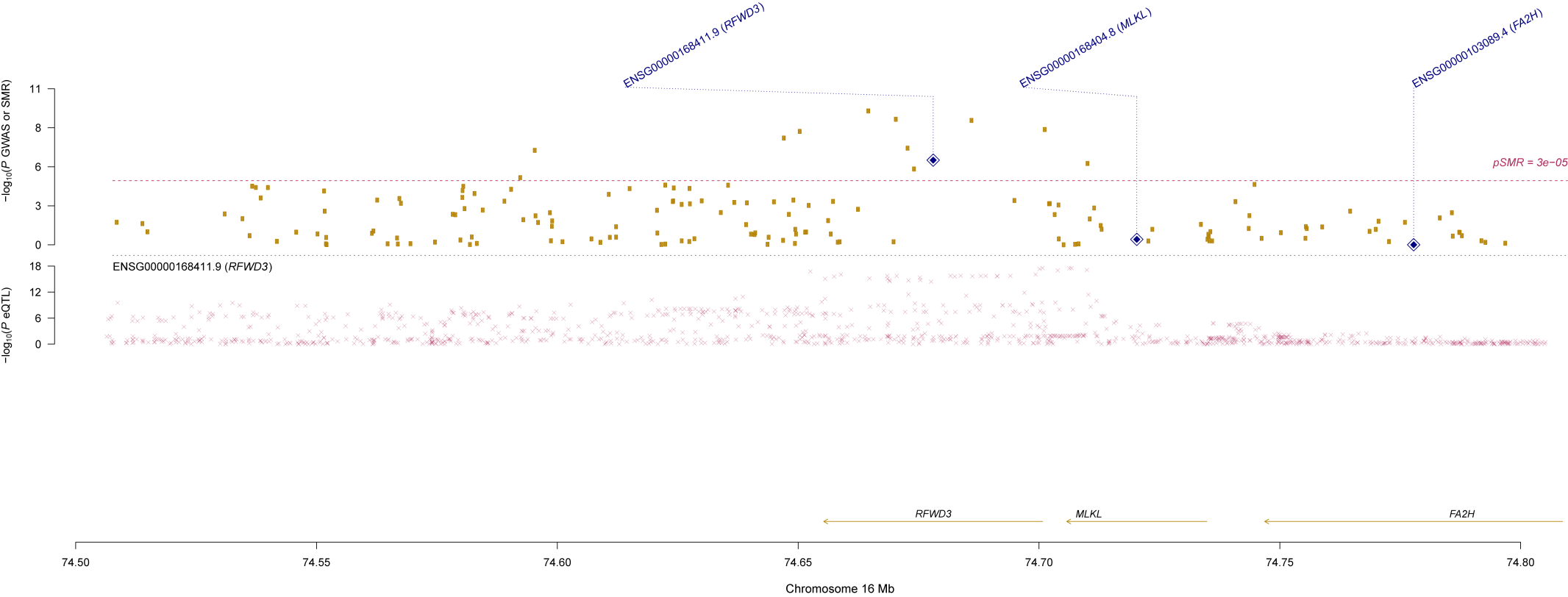


**Supplementary Figure 4d: Summary data-based Mendelian Randomization (SMR) analysis locus plot for multiple myeloma.** Upper panel - brown dots represent *P*-values for SNPs from the GWAS meta-analysis, diamonds represent *P-*values for probes from the SMR test; lower panel – crosses represent eQTL *P*-values of SNPs from whole blood with genes passing the SMR (i.e. *P_SMR_* < 0.001) and HEIDI (*i.e*. *P_HEIDI_* > 0.05) tests highlighted in red.

**
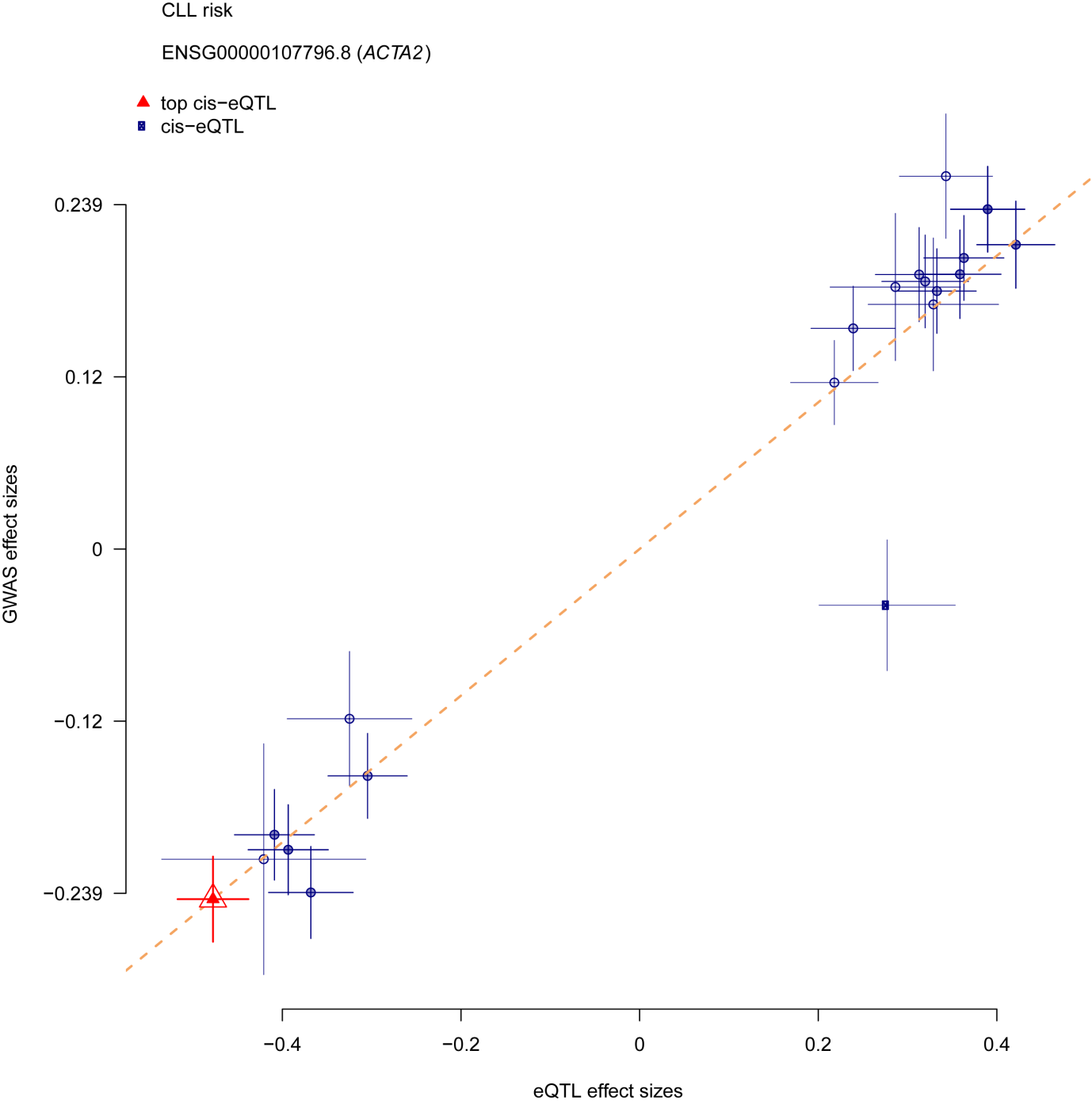
**

**Supplementary Figure 5a: Summary data-based Mendelian Randomization analysis effect plot for chronic lymphocytic leukaemia.** Blue dots represent effect sizes of SNPs from the GWAS meta-analysis against those from the eQTL study of whole blood. The top *cis*-eQTL is highlighted by a red diamond. Error bars are the standard errors of the SNP effects. An estimate of *b_xy_* at the top *cis*-eQTL is represented by the orange dotted line.

**
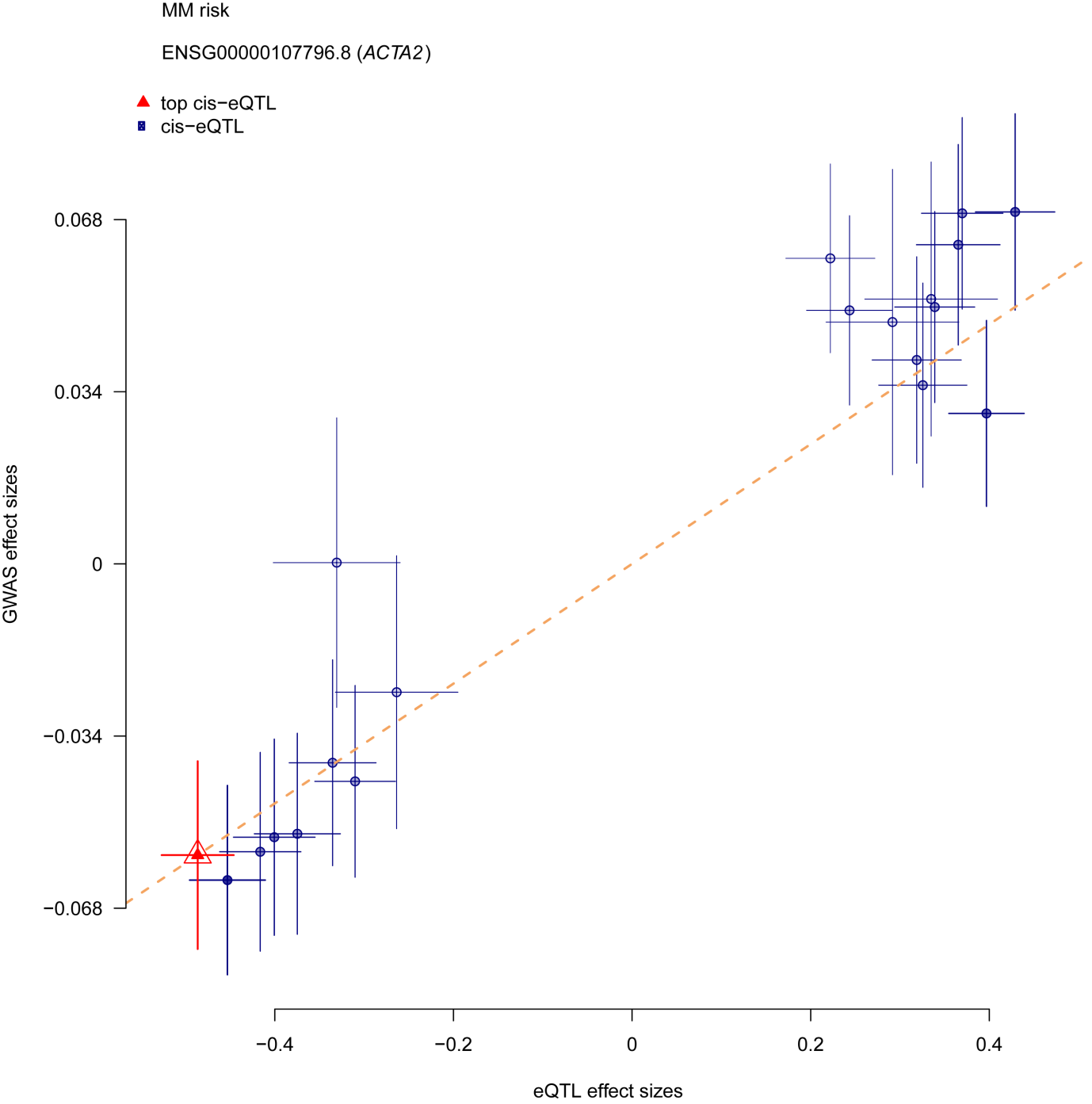
**

**Supplementary Figure 5b: Summary data-based Mendelian Randomization analysis effect plot for multiple myeloma.** Blue dots represent effect sizes of SNPs from the GWAS meta-analysis against those from the eQTL study of whole blood. The top *cis*-eQTL is highlighted by a red diamond. Error bars are the standard errors of the SNP effects. An estimate of *b_xy_* at the top *cis*-eQTL is represented by the orange dotted line.

**
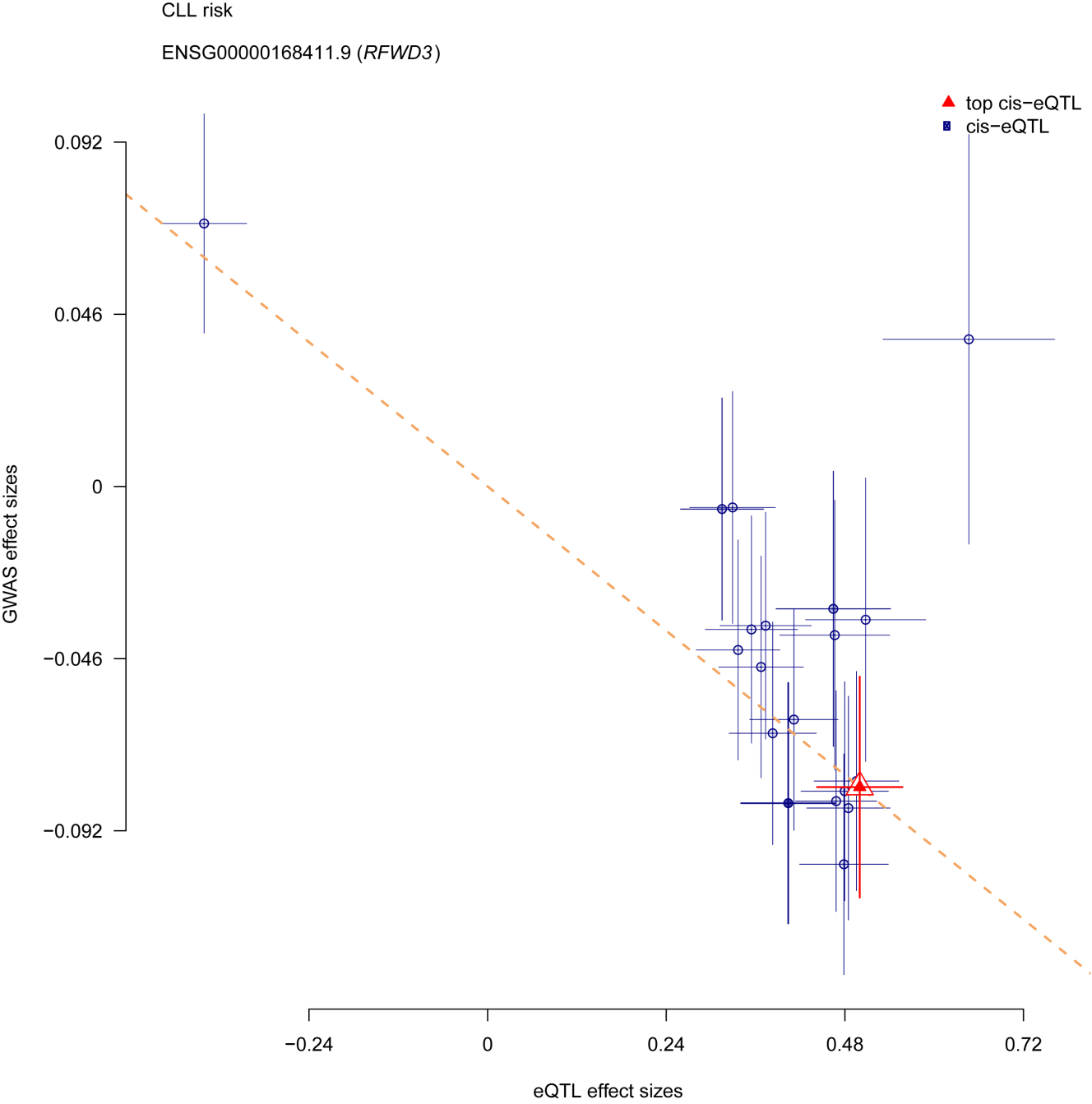
Supplementary Figure 5c: Summary data-based Mendelian Randomization analysis effect plot for chronic lymphocytic leukaemia.** Blue dots represent effect sizes of SNPs from the GWAS meta-analysis against those from the eQTL study of whole blood. The top *cis*-eQTL is highlighted by a red diamond. Error bars are the standard errors of the SNP effects. An estimate of *b_xy_* at the top *cis*-eQTL is represented by the orange dotted line.

**
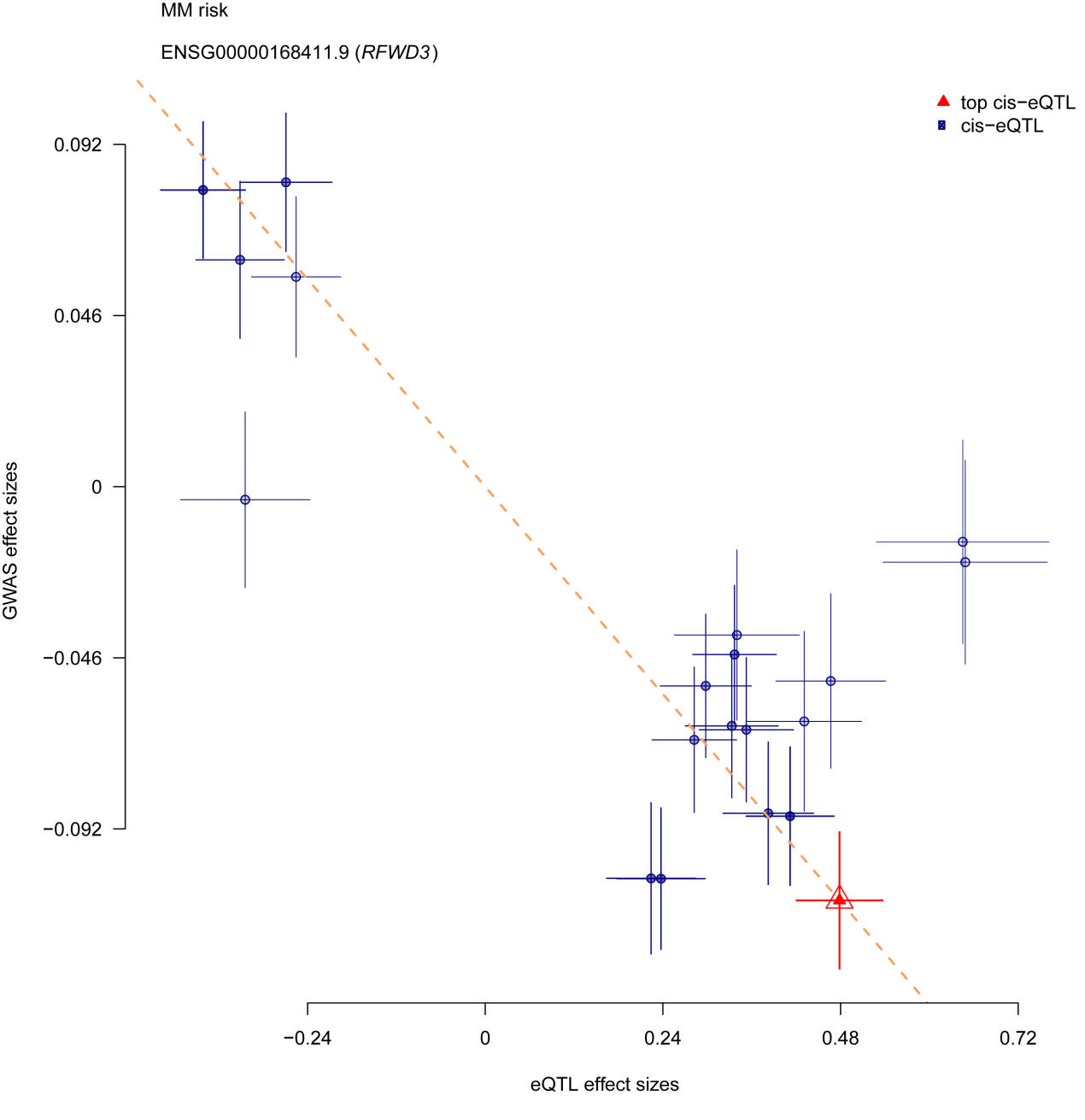
Supplementary Figure 5d: Summary data-based Mendelian Randomization analysis effect plot for multiple myeloma.** Blue dots represent effect sizes of SNPs from the GWAS meta-analysis against those from the eQTL study of whole blood. The top *cis*-eQTL is highlighted by a red diamond. Error bars are the standard errors of the SNP effects. An estimate of *b_xy_* at the top *cis*-eQTL is represented by the orange dotted line.

**REFERENCES**

1. Finucane, H.K. *et al.* Partitioning heritability by functional annotation using genome-wide association summary statistics. **47**, 1228-35 (2015).
